# Translation and Validation of the Questionnaire on Acceptance to Telemedicine from the Technology Acceptance Model (TAM) for Use in Malaysia

**DOI:** 10.1155/2022/9123887

**Published:** 2022-04-20

**Authors:** Masliyana Husin, Norazida Ab Rahman, Mohamad Adam Bujang, Sock Wen Ng, Kawselyah Juval, Wen Yea Hwong, Sheamini Sivasampu

**Affiliations:** ^1^Institute for Clinical Research, National Institutes of Health, Ministry of Health Malaysia, No. 1 Jalan Setia Murni U13/52, Seksyen U1340170 Shah Alam, Selangor, Malaysia; ^2^Clinical Research Centre, Sarawak General Hospital, Jalan Hospital, 93586 Kuching, Sarawak, Malaysia; ^3^Family Health Development Division, Ministry of Health Malaysia, Level 8 & 9, Block E10, E Complex, 62590 Putrajaya, Malaysia; ^4^Julius Center for Health Sciences and Primary Care, University Medical Center Utrecht, Utrecht University, Utrecht, Netherlands

## Abstract

The COVID-19 pandemic has accelerated implementation of telemedicine in healthcare facilities for delivery of care. Healthcare providers' acceptance of the telemedicine services is important for successful implementation of this new system. A questionnaire based on the Technology Acceptance Model (TAM) has been used to measure user acceptance of telemedicine service. The aim of this study was to translate and validate the English version of the questionnaire into Malay, to extend the availability and utilization of this questionnaire in Malaysia. A forward and backward translation of the questionnaire was conducted to produce the TAM in the Malay version (Malay-TAM). Panel experts assessed content validity. Internal consistency reliability was determined using Cronbach's alpha. Confirmatory factor analysis based on structural equation modelling was performed to validate the factor structure. The questionnaire was then tested on and completed by 149 healthcare workers from several public health clinics across Malaysia. The Malay-TAM demonstrated good reliability with Cronbach's alphas ranging from 0.823 to 0.912. Factor analysis showed good convergent validity but relatively poor discriminant validity. All five constructs were retained to preserve content validity. The findings suggest that the Malay-TAM can serve as a reliable and valid instrument to measure acceptance to telemedicine.

## 1. Introduction

Digital service provision in the healthcare sector has met with a sudden increase due to the COVID-19 pandemic response [1]. Telemedicine initiatives in the form of facility-based synchronous video consultation between healthcare providers and patients which was previously introduced to increase accessibility and reduce cost [2] have now become almost mandatory in order to minimise risk of exposure to COVID-19 for patients and healthcare providers while continuing routine clinical visit [3]. In developed countries, change in governance and relaxation of regulations from professional and regulatory agencies in conducting video consultation have been observed during this public health emergency [4]. Evaluation of telemedicine has found it to be convenient to patients [5, 6], improve their intermediate outcome [7], and be cost-effective [8] and cost-saving [9].

Although many potential benefits were seen when telemedicine is used, adoption and uptake in the real world have been slow because of difficulties with implementation [10]. Factors such as incentives and policies, infrastructure, organisational readiness, engagement of healthcare providers, healthcare providers' knowledge and beliefs, and relevance to workflows, processes, and systems are critical to implementation [11]. Acceptance is a perceptual phenomenon where new experiences on a system are evaluated before decisions to use are made based on the benefits and limitations of that experience. They are influenced by the users' knowledge and beliefs, and it determines their adoption. Understanding and measuring a system's acceptance allow for better design and prediction of the response of the users to the new system [12]. Studies have shown that the increase in healthcare providers' acceptance toward telemedicine also increases its implementation [11].

In order to supplement the evaluation of telemedicine implementation, a scoping review showed that many studies have assessed healthcare provider's acceptance using validated measurements such as [13], one of which is utilising the Technology Acceptance Model (TAM). The proposed theoretical framework is adapted from Kissi et al.'s [14] model of telemedicine acceptance and comprises of five constructs: perceived usefulness (PU), perceived ease of use (PEOU), behavioral intention (BI), attitude toward using and actual use (ATU), and user satisfaction (SE). Perceived usefulness is defined as the degree to which a person believes that using telemedicine services would enhance his or her job performance. Perceived ease of use is defined as the degree to which a person believes that using telemedicine services will be free of effort [15]. Behavioral intention is a measure of the intention to use motivated by one's behavior [16], and ATU defines the use of the telemedicine service in healthcare centers. User satisfaction is defined as the level of satisfaction in the use of telemedicine services. The model denotes effect of PU and PEOU to ATU and SE through BI in agreement with what TAM postulates [14, 16].

The Technology Acceptance Model (TAM) was developed to measure how well a technology “fits” with user tasks [15]. It is currently the most widely applied and empirically tested model in vast areas and the study setting [17, 18] and has been translated to many different languages such as Arabic [19], French [20], and Chinese [21, 22]. This model has been shown to be capable to explain about 40% of information technology (IT) use [23]. TAM is able to predict the acceptance of IT in which the intention to use and behavior intention of individuals are affected by two variables: perceived ease of use and perceived usefulness. When empirically tested to the field of telemedicine, studies across the world have found that perceived usefulness strongly predicts the behavioral intention more than perceived ease of use [17].

Measurement of acceptance using TAM has focused on the acceptance of health informatics [18] with only limited number of studies on videoconferencing or telemedicine [22, 24].

Most of the studies tried to understand perspective among healthcare providers in comparison to patients. In the studies that involved patients, perceived usefulness was found to have significant effects on telemedicine acceptance [22]. Among the studies on healthcare providers, perceived usefulness was also a strong determinant in physician-to-physician and physician-to-specialist remote consultation and referral in the Malaysian setting [24]. Another local study [25] found that other than perceived usefulness, moderators such as government policies, top management support, and computer self-efficiency affect telemedicine acceptance by healthcare providers in public hospitals.

Of yet, there is still an absence of a psychometrically validated TAM measurement tool in the Malay, the national language of Malaysia. Thus, the aim of our study is to validate the TAM questionnaire into the Malay language and examine the reliability and validity and test the relationship between the constructs for the translated Malay-TAM questionnaire. This validated tool will be used as part of the effort to understand the extent of acceptance for telemedicine service among local healthcare providers, in the attempt to guide implementation of such service in Malaysia.

## 2. Methods

We adapted the TAM questionnaire that utilises an extended TAM model and consists of five domains, 4 items for each domain totaling up to 20 items. The items were measured using a 5-point Likert scale ranging from “strongly agree” to “strongly disagree.” The scoring method is by averaging the total summation for each item.

### 2.1. Validation Process

#### 2.1.1. Content Validity

Content validity was assessed through literature review and judgement from panel experts that consist of medical doctors, pharmacist, public health specialists, and epidemiologist. All experts had experience working in primary care or district health office. Comparisons between different instruments available in the literature to the adapted instrument were made. Amendments were made based on the experts' feedback. In general, all items were scientifically relevant and acceptable.

#### 2.1.2. Translation

The questionnaire was translated by forward and backward translation in accordance with the World Health Organization's 2013 [26] recommendation after obtaining permission from the original author of the questionnaire. Translation of the original English instrument into the Malay language (forward translation) was conducted independently by two individuals who are bilinguals with Malay as their mother tongue. T1 is a medical doctor, and T2 is a university student majoring in the biomedical field. T1 was aware of the concepts being examined while T2 was not. The two forwarded-translated versions were reviewed by a third individual who is also fluent in both languages, and discrepancies between the terms were resolved through consensus discussions. The harmonised version was subsequently sent to two other independent bilingual individuals (BT1 and BT2) for back translation into English. BT1 is a pharmacist who has used Malay and English in her work for more than 10 years. BT2 is a medical doctor from a research institute who is fluent in both Malay and English.

Both BT1 and BT2 had no exposure to the original English questionnaire. The Malay and English translated versions were then compared with the original version by the research team to ensure conceptual and cultural equivalence. Ambiguities and discrepancies were discussed and resolved, and a prefinal version was prepared.

#### 2.1.3. Face Validity and Cognitive Debriefing

The prefinal translated version questionnaire was then administered to seven healthcare providers that were involved in the pilot Virtual Clinic Program at public primary healthcare clinics. The Virtual Clinic Program is a planned, synchronous video consultation between healthcare providers and patients. This service was initiated in five proof-of-concept clinics in June 2019 and has since been expanded to 35 more clinics. The service utilises a video consultation application platform. After completing the questionnaire, each participant was asked to elaborate what they thought each questionnaire item and their corresponding response meant. They were also asked whether there were any items in the questionnaire that were difficult to understand and to provide suggestions on how to make it clearer. This approach allowed the investigators to determine its comprehensibility and clarity while ensuring that the translated items retained the meaning of the original items. The research team discussed the comments and made appropriate amendments.

#### 2.1.4. Pilot Study

The pilot study was conducted in another group of 30 healthcare providers from the target population. No further modifications were required after the cognitive debriefing. We assessed the reliability of the questionnaire, and once Cronbach's alpha was satisfactory, we proceeded with the validation.

#### 2.1.5. Validation Field Study

A cross-sectional study was conducted between September and October 2020 during the COVID-19 pandemic when healthcare providers in public primary healthcare clinics had to adapt to the new norm of consulting patients remotely in order to manage their diseases. We included respondents whom are both users and nonusers of the Virtual Clinic Program to emulate the original paper. The research tool was disseminated via an online survey that can be accessed through a QR code link that was displayed at the beginning and at the end of every training session during the Virtual Clinic Program training. The self-administered questionnaire was filled by respondents which consisted of family medicine specialists, medical officers, nurses, pharmacists, allied health officers, and information technology officers who were part of the team involved in the provision of virtual clinic services at their respective primary care clinics. Based on the recommended minimum sample size of five subjects per item and the questionnaire that comprised of 20 items, a minimum requirement of 100 participants was set for this study [27]. Convergent validity and discriminant validity were used to measure construct validity. Convergent validity is how close and statistically related the items are with the proper constructs whereas discriminant validity is how different and unrelated the items are within a construct to items from another construct. Confirmatory factor analysis (CFA) was used to measure convergent and discriminant validity.

### 2.2. Statistical Analyses

Structural equation modelling (SEM) that consists of CFA and path analysis was performed using the “Lavaan” package in R software [28]. Model identification (latent variable estimation) was performed using a variance standardization method. Discriminant validity was established at the construct level using the comparison of square root of the average variance extracted to shared variance (AVE-SV criterion) proposed by Fornell and Larcker [29].

Model evaluation based on the fit indices for the test of a single path coefficient was calculated and assessed using these parameters with these cut-off values: chi‐square test (*χ*^2^) < 3.0, comparative fit index (CFI) > 0.90, root mean square error of approximation (RMSEA) < 0.08, and standardized root mean square residual (SRMR) < 0.08 [30].

## 3. Results

A total of 160 questionnaires were retrieved from participants; however, 11 were excluded as they were responses from unintended individuals and 149 responses were included in analysis giving a response rate of 93.2% participation. The respondents consisted largely of medical officers and family medicine specialists, followed by assistant medical officers, information technology officers, nurses and healthcare administrators. Pharmacists and allied health officers accounted for less than 5% each. There were 68 (45.6%) males and 81 (54.3%) females among the respondents. Out of the 149 responses, 55 have had exposure while 94 indicated that they have not been exposed to telemedicine service before.  Table 1 shows the description of the respondents.

Factor loading (FL), average variance extracted (AVE), and construct reliabilities (CR) are presented in Table 2. The FL for the PU construct ranges from 0.803 to 0.905, -0.539 to 0.839 for PEOU, 0.583 to 0.881 for BI, 0.708 to 0.813 for ATU, and 0.759 to 0.884 for SE. All items except for PEOU 3 and PEOU 4 reached the cut-off level of above 0.500 as recommended by Igbaria et al. [31]. The average variance extracted also showed a value more than 0.500 except for the PEOU construct. This fulfilled the convergent validity whereby items are related statistically with the proper constructs based on theoretical foundations, with the exception for PEOU.

When looking at each construct, Cronbach's alpha coefficient for PEOU was -0.123, which was low while the rest of Cronbach's alpha coefficient ranged from 0.823 to 0.912. The construct reliability exceeded the recommended threshold of 0.700 except for the PEOU construct. When PEOU 1 and PEOU 2 were deleted, Cronbach's alpha for PEOU was 0.200. When PEOU 3 and PEOU 4 were deleted, Cronbach's alpha for PEOU improved to 0.800, CR to 0.860, and AVE to 0.766 (not shown in table). This shows that the low Cronbach's alpha coefficient for the PEOU construct was due to items PEOU 3 and PEOU 4.  Table 2 shows the measurements and confirmatory factor analysis for the original model.

Based on the CFA findings, we measured the model fit for two different models as shown in Table 3. The model fit based on the original study showed a lower CFI of 0.896 with RMSEA and SRMR higher than the recommended value of 0.080. In the other model, where the items PEOU 3 and PEOU 4 were omitted, the indices were closer to the recommended values, and these indicated a better model fit.

Discriminant validity was assessed to ensure that the reflective constructs differed from each other. Based on the recommendation, the measurement items on their assigned latent variables should have an order of magnitude larger than their loadings on other variables. The correlations between items in any two constructs should be lower than the square root of the average variance shared by items within a construct. As shown in Table 4, the square root of the variance shared between a construct and its items (appearing in bold along the diagonal) was only greater between construct PEOU to PU and BI in the original model and after the two low loading items were deleted (not shown).

**Figure 1:**
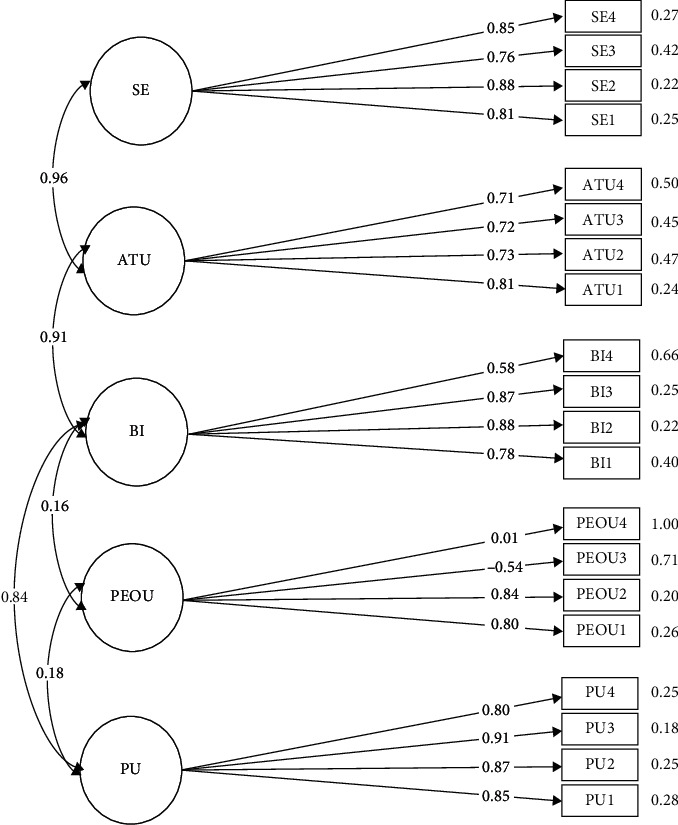
Causal path diagram for the original model.

The structural model for the original model showed significant positive relationships for all the constructs at differing significance as shown in Figure 1. PU (0.847, *p* < 0.001) suggested a positive and significant influence on BI. PEOU to PU (0.182, *p* < 0.05). PEOU did not correlate to BI (0.162, *p* = 0.08) while BI positively affected ATU (0.912, *p* < 0.001) and ultimately ATU to SE (0.963, *p* < 0.001). When the two items from PEOU were deleted, the structure showed a correlation of the PEOU construct to PU (0.219, *p* < 0.05) and to BI (0.173, *p* < 0.05).  Table 5 shows the relationship between each of the constructs for the original model. Figure 1 shows the path diagram for the original model and path diagram to the modified model can be seen in Supplementary Materials (Appendix A (available here)).

## 4. Discussion

This first attempt to translate and subsequently validate the TAM model in our local setting has demonstrated a high reliability in four out of five constructs. Similarly, items from four out of five constructs were highly correlated within their respective construct. The construct PEOU however showed poor reliability and validity.

### 4.1. Reliability

The reliability analysis shows that internal consistency of Malay-TAM was acceptable for four out of five constructs, which are the PU, BI, ATU, and SE. The high reliability in the four constructs is consistent with other studies that tested TAM in the Malaysian population [24, 25]. The low reliability in the PEOU construct could be contributed to its mixed format within the four items. In the English version, the first two items in the PEOU construct were negatively worded. PEOU 1 holds a statement about the technology being not clear and not understandable (“Telemedicine services are rigid and not flexible to interact with”) while PEOU 2 holds a statement about slow response time (“interacting with telemedicine services is often frustrating”). Constructs with a mixed-format may have made it challenging for respondent to retrieve the information and could potentially create confusion if they did not pay attention to the items which can interfere with internal consistency [32–35].

### 4.2. Convergent Validity

Construct validity was measured using convergent and discriminant validity. The convergent validity for four out of five constructs was above the recommended cut-off and showed that items within the PU, BI, ATU, and SE correlated highly with the constructs. Low correlation was seen in PEOU 3 (“Telemedicine services do not require several training to effectively use”) and PEOU 4 (“Telemedicine services are compatible with the existing clinical workflow”) within the PEOU construct. We postulate that the low correlations between items are due to the fact that learning and compatibility of the technology might not be seen as indicators for ease of use among our respondents. For the current study, respondents completed the questionnaire during the COVID-19 pandemic whereby conversion of clinic visits to virtual consultation platforms were happening rapidly. The respondent might feel that the amount of training given on the platform does not make it easier for them to carry out the actual service. This goes to show that the perspective of ease of use extends beyond solely understanding how to operate the platform. Similar findings have been reported by Hu et al. [36].

### 4.3. Discriminant Validity

Additionally, we also used discriminant validity to establish construct validity. When the square root of AVE was compared with the correlation coefficient, items within a construct were assessed if these items explain more variance than do the items of the other constructs. We found that discriminant validity only holds between PEOU to PU and BI. Meanwhile, the constructs PU to BI, BI to ATU, and ATU to SE showed low discriminant validity which is inconsistent with the findings on discriminant validity by the original author [14]. We postulate that this could be due to the different settings under which the telemedicine technology was used. Our respondents were healthcare providers who were familiarising with the telemedicine technology in order to reduce the clinic congestions in the times of the COVID-19 pandemic. In these settings, it was imperative for the healthcare providers to utilise telemedicine to ensure that patients were being followed-up as the face-to-face clinic visit has to be reduced in order to prevent the spread of COVID-19. Hence, this might have caused a skewness of the responses towards the right in which most respondents tend to have highly positive perception that telemedicine is useful (PU), report that they would highly use the technology (BI), report that they see the benefit when they use the technology (ATU), and perceive high satisfaction once they use the technology (SE) regardless of their skill levels (PEOU). We believe that the crucial need to use telemedicine affected the behavior of the respondents and potentially resulted in skewness towards the higher scores on the Likert scale, thus reducing variability in the responses. In other words, the poor discriminant is not likely due to poor items or construct as we adapted the items from a model that has been successfully validated multiple times and translated it using an established method. On top of that, our respondents' characteristics were also almost similar compared to the original study. The fact that the low discriminant validity only involves four constructs and spares the construct PEOU also points to the fact that the overlap in the construct could be sample and setting dependent [37]. This could suggest that if we apply the questionnaire before the COVID-19 pandemic, there is always the chance that the structure will hold as previously demonstrated by Kissi et al. [14].

### 4.4. Structural Path

In terms of the structural path, our findings on PEOU are in agreement with other studies [17]. The function of PEOU in determining BI has been debatable. Davis [15] who is the original author of TAM also did not find a linkage between PEOU and BI in his initial study. Keil et al. found that PU is more important than PEOU in determining actual use, and their study did not find improvement in PEOU despite improving the technology's user interface [38]. Hu et al. found PEOU to have low explanatory power to ATU relative to PU in users with high competence such as physicians. They attributed this to the fact that the usefulness of a technology outweighs the difficulty in operating the system as they can assimilate new technologies quickly if they know that it is beneficial for their patients [36]. The effect of PEOU is also evident in our structural path whereby there is no relationship between PEOU and BI before modifications. The correlations were only established once deletions of a few items were made in PEOU.

This study presented four model fit indices where three indicators met the requirement with one that can be considered borderline. In light of these findings, although there is low discriminant validity among four constructs and inconsistencies in the construct PEOU, we propose to retain the original model with five constructs based on its strong content validity. As mentioned, the low discriminant validity and the inconsistencies in the effect of PEOU are sample and setting dependent. Several authors found that PEOU is a relevant construct in the early stages of learning but becomes less important with time and experience [15, 39, 40]. Although it has low explanatory power when tested in the current sample of experienced and highly intelligent healthcare providers, this could change when the tool is administered on a different sample such as students or patients. It is imperative to understand the demographics of the study' respondents in depth taking into account the different levels of experience.

The strength of the study is that we tested the psychometric property of the established TAM model focusing on highly relevant criterion and examined the factor association within a sample of healthcare providers. The limitation is that the validity might not be generalisable to other population, the timing of the study falls within the COVID-19 pandemic, and the sample selection did not discriminate between different levels of respondents' experience. Additionally, the adapted tool measured perception of use and not objective use which could have caused reporting bias.

## 5. Conclusion

The Malay-TAM consisting of five constructs assessed through 18 items has good reliability and exhibits convergent validity but attenuated discriminant validity, possibly attributed to the change behavior among the respondents due to the COVID-19 pandemic. The tool is acceptable in measuring acceptance to telemedicine in the Malaysian population. In order for future studies to utilise the model with five constructs, a further relook into the constructs can be performed and PEOU 3 and PEOU 4 should undergo deletion with all PEOU items kept in a positive direction.

For future studies, this study recommends the questionnaire to be validated in different target populations to look into the constructs and PEOU items to be worded in a positive direction as shown in Supplementary Materials (Appendix A (available here)).

## Figures and Tables

**Table 1:** Respondent demographics.

Characteristics	Total (*N* = 149)	
	*n*	%
Gender		
Male	68	45.6%
Female	81	54.3%
Role		
Medical officers	37	24.8%
Family medicine specialist	31	20.8%
Assistant medical officer	29	19.4%
Information technology officer	20	13.4%
Nurse	16	10.7%
Healthcare administrator	10	6.7%
Pharmacist	3	2.0%
Nutritionist	1	0.6%
Others	2	0.1%
Previous experience with telemedicine service		
Yes	55	36.9%
No	94	63.1%

Others: temporary research officers.

**Table 2:** Measurements and confirmatory factor analysis for the original model.

Construct	Items	Unstandardized estimate	Standard error	Critical ratio	Standardized factor loadings	*p* value	Average variance extracted	Construct reliability	Cronbach alpha
PU	PU1	0.660	0.052	12.720	0.850	<0.001	0.734	0.917	0.912
	PU2	0.662	0.050	13.112	0.866	<0.001			
	PU3	0.730	0.052	14.118	0.905	<0.001			
	PU4	0.736	0.063	11.664	0.803	<0.001			

PEOU	PEOU1	0.816	0.085	9.627	0.802	<0.001	0.411	0.368	-0.123
	PEOU2	0.746	0.074	10.062	0.839	<0.001			
	PEOU3	-0.611	0.095	-6.448	-0.539	<0.001			
	PEOU4	0.054	0.073	0.748	0.067	<0.001			

BI	BI1	0.527	0.048	11.000	0.775	<0.001	0.617	0.862	0.828
	BI2	0.661	0.049	13.392	0.881	<0.001			
	BI3	0.724	0.055	13.057	0.867	<0.001			
	BI4	0.602	0.080	7.556	0.583	<0.001			

ATU	ATU1	0.621	0.052	11.825	0.813	<0.001	0.554	0.832	0.823
	ATU2	0.545	0.054	10.175	0.731	<0.001			
	ATU3	0.562	0.056	10.013	0.722	<0.001			
	ATU4	0.764	0.078	9.752	0.708	<0.001			

SE	SE1	0.640	0.054	11.755	0.808	<0.001	0.685	0.896	0.896
	SE2	0.720	0.053	13.551	0.884	<0.001			
	SE3	0.643	0.060	10.732	0.759	<0.001			
	SE4	0.666	0.052	12.801	0.854	<0.001			

PU: perceived usefulness; PEOU: perceived ease of use; BI: behavioral intention; ATU: attitude toward using and actual use; SE: user satisfaction.

**Table 3:** Fit of the overall model and revised model.

Model fit index	Recommended value	Overall results	Results if PEOU 3 and PEOU 4 are omitted
Chi-square/degree of freedom	<3.00	2.813	2.439
Comparative fit index	>0.90	0.869	0.913
Root-mean square error of approximation	<0.08	0.108	0.098
Standardized root-mean square residual	<0.08	0.149	0.059

PEOU: perceived ease of use.

**Table 4:** Discriminant validity for the original model.

Construct	PU	PEOU	BI	ATU	SE
PU	**0.857**				
PEOU	0.182^∗^	**0.641**			
BI	0.848^∗∗∗^	0.162	**0.785**		
ATU	0.902^∗∗∗^	0.049	0.913^∗∗∗^	**0.744**	
SE	0.829^∗∗∗^	0.062	0.881^∗∗∗^	0.964^∗∗∗^	**0.827**

PU: perceived usefulness; PEOU: perceived ease of use; BI: behavioral intention; ATU: attitude toward using and actual use; SE: user satisfaction. ^∗^*p* value < 0.05, ^∗∗^*p* value < 0.01, and ^∗∗∗^*p* value < 0.001.

**Table 5:** Structural model results for the original model.

Hypothesis	Path	Unstandard estimate	Standard error	Standard estimate	*p* value	Findings
H1	PU − >BI	0.847	0.032	0.847	<0.001	Supported
H2	PEOU − >BI	0.162	0.093	0.162	0.08	Not supported
H3	PEOU − >PU	0.182	0.091	0.182	<0.05	Supported
H4	BI − >ATU	0.912	0.029	0.912	<0.001	Supported
H5	ATU − >SE	0.963	0.022	0.963	<0.001	Supported

PU: perceived usefulness; PEOU: perceived ease of use; BI: behavioral intention; ATU: attitude toward using and actual use; SE: user satisfaction.

## Data Availability

The data presented in this study are available on request from the corresponding author.

## Supplementary Materials

Supplementary Materials The structural path diagram for the modified TAM model (Appendix A). Part B of the questionnaire titled “Predictive factors of physicians' satisfaction with telemedicine services acceptance in Ghanaian health facilities” with the final translated version in Malay including items to be worded in a positive direction (Appendix B).
