# Supplementary material for: Translation and Validation of the Questionnaire on Acceptance to Telemedicine from the Technology Acceptance Model (TAM) for Use in Malaysia
Source: Biomed Res Int. 2022 Apr 20;2022:9123887. doi: 10.1155/2022/9123887 (PMC9020140; doi:10.1155/2022/9123887)
Supplement: Supplementary Materials — The structural path diagram for the modified TAM model (Appendix A). Part B of the questionnaire titled “Predictive factors of physicians' satisfaction with telemedicine services acceptance in Ghanaian health facilities” with the final translated version in Malay including items to be worded in a positive direction (Appendix B). [file 9123887.f1.docx]

# SUPPLEMENTARY MATERIAL

# Appendix A

#
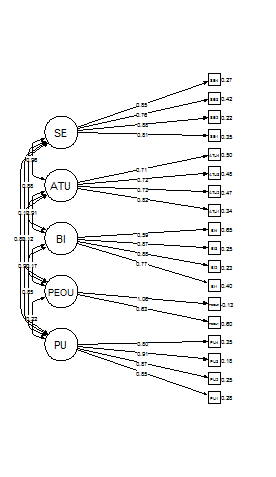


**Figure 1: Causal path diagram for the modified model**

# Appendix B

**A Predictive factors of physicians’ satisfaction with telemedicine services acceptance in Ghanaian health facilities with Malay translation**

# PART B

Please indicate to what extent you agree or disagree with the following statements on Predictive factors of physicians’ satisfaction with telemedicine services acceptance in Ghanaian health facilities by circling the appropriate number/scale.

# (1 = Strongly Disagree; 2 = Disagree; 3 = Neutral; 4 = Agree; 5 = Strongly Agree)

***(1 = Sangat Tidak Setuju; 2 = Tidak Setuju; 3 = Neutral; 4 = Setuju; 5 = Sangat Setuju)***

|  | Question | Responses | | | | |
| --- | --- | --- | --- | --- | --- | --- |
| **Attributes of telemedicine services (Perceived Usefulness)**  ***Ciri-ciri perkhidmatan teleperubatan (persepsi kegunaan)*** | | | | | | |
| 1 | Using telemedicine service improves my job effectiveness  and performance.  *Penggunaan perkhidmatan teleperubatan meningkatkan keberkesanan tugas dan prestasi saya* | 1 | 2 | 3 | 4 | 5 |
| 2 | Using telemedicine service gives me greater control over my  work.  *Penggunaan perkhidmatan teleperubatan memberikan saya lebih kawalan terhadap kerja saya* | 1 | 2 | 3 | 4 | 5 |
| 3 | Telemedicine service enables me to accomplish tasks more  quickly and makes me more productive.  *Perkhidmatan teleperubatan membolehkan saya melaksanakan tugas dengan lebih pantas dan menjadikan saya lebih produktif* | 1 | 2 | 3 | 4 | 5 |
| 4 | Telemedicine services help to get current diagnosis and  treatment plans for patients.  *Perkhidmatan teleperubatan membantu bagi mendapatkan diagnosis terkini dan pelan rawatan untuk pesakit* | 1 | 2 | 3 | 4 | 5 |

| **Attributes of telemedicine services (Perceived Ease of Use)**  ***Ciri-ciri perkhidmatan teleperubatan (persepsi kemudahan penggunaan)*** | | | | | | | |
| --- | --- | --- | --- | --- | --- | --- | --- |
| 1 | Telemedicine services are rigid and not flexible to interact  with.  *Teleperubatan merupakan servis yang tegar dan tidak fleksibel untuk berinteraksi* | 1 | 2 | 3 | 4 | | 5 |
| 2 | Interacting with telemedicine services is often frustrating.  *Interaksi dengan perkhidmatan teleperubatan selalunya mengecewakan* | 1 | 2 | 3 | 4 | | 5 |
| 3 | Telemedicine services do not require several training to  effectively use.  *Perkhidmatan teleperubatan tidak memerlukan beberapa siri latihan untuk digunakan secara berkesan* | 1 | 2 | 3 | 4 | | 5 |
| 4 | Telemedicine services are compatible with the existing clinical workflow.  *Perkhidmatan teleperubatan adalah serasi dengan aliran kerja klinikal yang sedia ada* | 1 | 2 | 3 | 4 | | 5 |
| **Individual Behavioral Intention to telemedicine services adoption and use**  ***Niat tingkah laku individu untuk menerima dan menggunakan perkhidmatan teleperubatan*** | | | | | | | |
| 1 | I have a positive intention to adopt and use telemedicine services.  *Saya mempunyai niat positif untuk menerima pakai dan menggunakan perkhidmatan teleperubatan* | 1 | 2 | 3 | 4 | 5 | |
| 2 | Telemedicine services positively support the treatment plan of my patients.  *Perkhidmatan teleperubatan menyokong pelan rawatan pesakit-pesakit saya secara positif* | 1 | 2 | 3 | 4 | 5 | |
| 3 | Telemedicine service provides me with a more  comprehensive care service.  *Perkhidmatan teleperubatan menyediakan saya dengan perkhidmatan penjagaan yang lebih komprehensif* | 1 | 2 | 3 | 4 | 5 | |
| 4 | I have gained rich and diverse experiences in telemedicine  services delivery.  *Saya telah memperoleh pelbagai pengalaman berharga dalam penyampaian perkhidmatan teleperubatan* | 1 | 2 | 3 | 4 | 5 | |
| **Actual telemedicine service used by Physicians**  ***Penggunaan sebenar perkhidmatan teleperubatan oleh pegawai*** | | | | | | | |
| 1 | Telemedicine is beneficial for my practice.  *Teleperubatan adalah bermanfaat kepada praktis saya* | 1 | 2 | 3 | 4 | 5 | |
| 2 | Using telemedicine services enables me to get into contact  with patients who seldom come to the hospital.  *Penggunaan perkhidmatan teleperubatan membolehkan saya untuk berhubung dengan pesakit yang jarang hadir ke klinik* | 1 | 2 | 3 | 4 | 5 | |
| 3 | Telemedicine service helps to take care of patients and  avoids several referrals.  *Perkhidmatan teleperubatan membantu bagi menjaga pesakit dan mengelakkan beberapa rujukan* | 1 | 2 | 3 | 4 | 5 | |
| 4 | Telemedicine helps to treat more patients with fewer  Clinicians.  *Teleperubatan membantu merawat lebih ramai pesakit dengan jumlah doktor yang lebih sedikit* | 1 | 2 | 3 | 4 | 5 | |
| **Physicians satisfaction with telemedicine services**  ***Kepuasan pegawai terhadap perkhidmatan teleperubatan*** | | | | | | | |
| 1 | Physicians feel comfortable communicating with patients  when using telemedicine service.  *Saya berasa selesa berkomunikasi dengan pesakit ketika menggunakan perkhidmatan teleperubatan* | 1 | 2 | 3 | 4 | 5 | |
| 2 | Physicians feel at ease in adding telemedicine services to  their existing clinical workflows.  *Saya berasa senang untuk menambah perkhidmatan teleperubatan ke aliran kerja klinikal yang sedia ada* | 1 | 2 | 3 | 4 | 5 | |
| 3 | Telemedicine services save physicians time for traveling far  for patients care delivery.  *Perkhidmatan teleperubatan menjimatkan masa saya dari melakukan perjalanan jauh untuk penyampaian penjagaan pesakit* | 1 | 2 | 3 | 4 | 5 | |
| 4 | Overall, physicians are satisfied with telemedicine services.  *Secara keseluruhan, saya berpuas hati dengan perkhidmatan teleperubatan* | 1 | 2 | 3 | 4 | 5 | |
| Proposed new items in positive direction | | | | | | | |
| **Attributes of telemedicine services (Perceived Ease of Use)**  ***Ciri-ciri perkhidmatan teleperubatan (persepsi kemudahan penggunaan)*** | | | | | | | |
| 1 | I think I could easily learn how to use Telemedicine services  *Saya dapat belajar menggunakan perkhidmatan teleperubatan dengan mudah* | 1 | 2 | 3 | 4 | 5 | |
| 2 | I think it would be easy to perform the tasks necessary for the care of my patients using telemedicine services  *Saya berpendapat ianya mudah untuk melaksanakan tugas penjagaan pesakit*  *dengan menggunakan perkhidmatan teleperubatan.* | 1 | 2 | 3 | 4 | 5 | |
| 3 | I think I will find it easy to acquire the necessary skills to use telemedicine services  *Adalah mudah bagi saya untuk memperoleh kemahiran untuk menggunakan*  *perkhidmatan teleperubatan* | 1 | 2 | 3 | 4 | 5 | |
| 4 | I think that telemedicine services will be easy to use  *Saya mendapati perkhidmatan teleperubatan adalah mudah untuk digunakan* | 1 | 2 | 3 | 4 | 5 | |
